# Dual-Signal Direct Time-of-Flight Method for Long-Range Groundwater Level Monitoring in Observation Wells

**DOI:** 10.3390/s26123672

**Published:** 2026-06-09

**Authors:** Abror Shavkatovich Buriboev, Farkhat Rajabov, Jamoljon Djumanov, Khudoyorkhon Jamolov, Akmal Abduvaitov, Temur Azamov, Ilhom Rahmatullayev, Cheolwon Lee

**Affiliations:** 1 Department of Artificial Intelligence, Gachon University, Seongnam 13120, Republic of Korea; abror1989@gachon.ac.kr; 2 Department of Computer Systems, Tashkent University of Information Technologies Named After Muhammad Al-Khwarizmi, Tashkent 100200, Uzbekistan; radjabov@tuit.uz (F.R.); jamoljon@mail.ru (J.D.); xudoyorjamolov@gmail.com (K.J.); 3 Department of Information Technologies, Samarkand Branch of Tashkent University of Information Technologies Named after Muhammad Al-Khwarizmi, Samarkand 140100, Uzbekistan; abduvaitovakmal@gmail.com; 4 Department of Artificial Intelligence, Samarkand State University Named After Sharof Rashidov, Samarkand 140100, Uzbekistan; 5 Agency Innovative Development, Tashkent 100174, Uzbekistan; uziric@gmail.com; 6 Department of Exact Sciences, Kimyo International University in Tashkent, Tashkent 100121, Uzbekistan; ilhom9001@gmail.com; 7 Department of Computer Engineering, Konkuk University, Chungju 27478, Republic of Korea

**Keywords:** groundwater monitoring, observation wells, ultrasonic time-of-flight, direct acoustic propagation, 433 MHz ISM, LoRa synchronization, floating receiver, power gating, median filtering

## Abstract

Accurate and reliable groundwater-level monitoring in deep observation wells remains difficult for conventional non-contact ultrasonic systems because narrow tubular geometries intensify multipath reflections, signal attenuation, and echo ambiguity. This study proposes a dual-signal direct time-of-flight (ToF) method that combines radiofrequency (RF) synchronization with one-way airborne ultrasonic propagation to a floating receiver located at the groundwater surface. In the proposed architecture, the RF signal provides a near-instantaneous time reference, whereas the ultrasonic signal defines the propagation delay, thereby eliminating dependence on echo-based ranging. The system integrates a wellhead surface unit for synchronized transmission and control, a floating unit for ToF acquisition and embedded processing, and an optional reference channel for in situ estimation of the effective sound speed. A duty-cycled power architecture is used to support low-power long-term deployment, while a multi-shot acquisition strategy with a median-like estimator improves robustness against startup transients, timing jitters, and false detections. Field validation was conducted over a 12-month period under actual groundwater-monitoring conditions, during which the groundwater depth varied between 14 m and 30 m below the wellhead datum. Within this field-validation interval, the proposed system achieved a mean absolute error of 0.048 m, a maximum absolute error of 0.050 m, and an overall valid detection rate of 99.4% over 358 valid cycles out of 360 scheduled cycles. In addition, a separate range-dependent confined-tubular propagation test was conducted to evaluate the extended detection capability of the RF-synchronized one-way ultrasonic ToF architecture. This test demonstrated stable acoustic-link ToF detection up to 300 m inside the tested 170 mm confined plastic pipeline. Therefore, the 300 m result should be interpreted as a range-dependent valid-detection result rather than as a 12-month groundwater-depth validation over the full 300 m interval. These results demonstrate that the proposed direct-ToF method provides an RF-synchronized one-way ultrasonic ToF framework with a floating receiver for groundwater-level monitoring in deep observation wells, while remaining compatible with low-power and IoT-based environmental monitoring systems.

## 1. Introduction

Groundwater observation wells play a critical role in hydrogeological monitoring by providing direct insight into aquifer dynamics under climatic variability and increasing anthropogenic pressure. In many regions, including Central Asia, groundwater constitutes a substantial portion of potable water supply, making accurate and continuous monitoring essential for both scientific analysis and operational water management. Reliable groundwater level measurements support the assessment of recharge and discharge processes, seasonal variability, and long-term resource sustainability. However, observation wells are often distributed across remote areas with limited access to power and infrastructure, imposing strict requirements on measurement autonomy, robustness, and operational reliability [1,2,3].

Conventional groundwater monitoring systems commonly employ submerged pressure transducers installed below the water surface. These instruments are accurate, mature, and widely used in practical groundwater-monitoring applications. However, because they operate in continuous contact with groundwater, long-term deployment may require periodic cleaning, recalibration, cable inspection, and maintenance. In some field environments, fouling, sediment deposition, chemical contamination, corrosion, and mechanical stress on cables may contribute to measurement drift or operational degradation. These limitations do not reduce the importance of pressure-based instruments, but they motivate the investigation of alternative measurement configurations that reduce dependence on permanently submerged pressure-sensing elements.

Water-level measurement methods that reduce dependence on submerged pressure sensing typically employ ultrasonic or radar-based ranging. Echo-based ultrasonic systems are widely used due to their low cost and simplicity; however, in narrow well geometries they are highly sensitive to multipath reflections from casing walls, fittings, and surface disturbances. This significantly reduces measurement reliability and limits the effective range. Radar-based methods offer improved robustness under certain conditions, but their cost and installation constraints may hinder widespread deployment in large-scale monitoring networks. Existing studies and reviews highlight these trade-offs, particularly in applications where geometry and environmental conditions deviate from open-channel configurations [4,5,6].

In parallel, advances in IoT-based monitoring systems have enabled low-cost, distributed sensing networks with remote data transmission and long-term operation. Such systems typically emphasize communication efficiency and energy management but often rely on conventional sensing principles that remain sensitive to environmental and geometric constraints. In particular, accurate timing for acoustics ranging under aggressive du-ty-cycled operation remains a challenge in low-power deployments.

Despite these developments, a clear gap remains in measurement methods specifically tailored to deep, narrow observation wells, where reflection-based approaches suffer from ambiguity and limited range. To address this challenge, this study proposes a dual-signal direct time-of-flight (ToF) method that combines radiofrequency (RF) synchronization with one-way ultrasonic propagation. In the proposed approach, a 433 MHz ISM-band signal provides a near-instantaneous timing reference, while a 40 kHz ultrasonic signal propagates directly from the wellhead transmitter to a floating receiver located at the water surface. This configuration eliminates reliance on echo detection and significantly reduces errors associated with multipath reflections.

To enhance robustness and adaptability, the system incorporates an optional reference channel at a known distance to estimate the effective speed of sound under in situ environmental conditions. In addition, low-power duty-cycled architecture is implemented to enable long-term autonomous operation. To mitigate transient effects introduced by power cycling and RF front-end stabilization, each measurement cycle acquires multiple ToF samples and applies a median-like estimator to suppress outliers while maintaining computational efficiency.

The main contributions of this study can be summarized as follows:

- A dual-signal direct-ToF sensing method is proposed for deep observation wells, combining RF-based synchronization with one-way airborne ultrasonic propagation to a floating receiver, thereby avoiding echo ambiguity and reducing multipath-related errors in narrow tubular geometries.
- A low-power RF-synchronized one-way ultrasonic ToF architecture with a floating receiver is developed, integrating a wellhead surface unit, a floating receiving unit, optional sound-speed reference calibration, and a 10-shot median-like estimator to improve measurement stability under duty-cycled operation.
- The proposed system is evaluated through a 12-month field-validation campaign, achieving a mean absolute error of 0.048 m, a maximum absolute error of 0.050 m, and a valid detection rate of 99.4%. In addition, a separate range-dependent confined-tubular acoustic-link test demonstrated stable valid ToF detection up to 300 m inside the tested 170 mm plastic pipeline.

The remainder of this paper is organized as follows. Section 3 describes the proposed measurement method, system architecture, and synchronization strategy. Section 4 presents experimental validation results, including range performance and accuracy analysis. Section 5 discusses the physical interpretation, comparative context, and practical limitations of the proposed approach. Section 6 concludes the paper and outlines directions for future work.

## 2. Related Works

Groundwater monitoring has traditionally relied on contact-based sensing technologies, particularly submerged pressure transducers and data loggers. Regional studies by Zokirov K. and colleagues have emphasized the importance of groundwater resources for water supply and environmental sustainability, highlighting the need for reliable long-term monitoring systems [7]. In addition, early system-level developments by Turaev B., Yaxshiboev R., and co-authors explored mathematical models and remote monitoring devices for groundwater level assessment, demonstrating the importance of integrating sensing and communication subsystems [8]. More broadly, Saymanov et al. developed an online IoT-based groundwater monitoring system tailored for Central Asian conditions, illustrating how real-time data acquisition can support strategic water management decisions in remote and arid regions [9]. To overcome maintenance limitations associated with submerged sensors, non-contact measurement approaches have been widely investigated. Ultrasonic sensing remains one of the most widely used techniques due to its simplicity and low cost. For example, Panagopoulos Y. et al. evaluated an ultrasonic water stage monitoring sensor and showed that echo-based measurements are sensitive to environmental disturbances and installation conditions, particularly in confined environments [10]. Similarly, Kang S. and co-authors proposed an energy-efficient ultrasonic detection system based on duty-cycled operation, demonstrating improved energy performance but still relying on reflection-based ranging [11]. Bae and Ji further demonstrated that water level data acquired by ultrasonic sensors in stream-scale channels exhibit significant outlier rates arising from multipath reflections and wave disturbances, and proposed a robust outlier removal framework based on median absolute deviation estimation to improve measurement reliability [12]. A comprehensive review of ultrasonic ranging methods by Zhang et al. confirmed that echo-based systems require environmental compensation for temperature and humidity to maintain acceptable accuracy, and that signal processing strategies such as multi-sample averaging are essential when operating under variable acoustic conditions [13]. More recently, Guo et al. investigated the use of ultrasonic probes inside metal borehole casings, demonstrating that frequency selection must account for wall thickness and internal geometry to minimize interference losses and achieve reliable signal transmission through confined tubular structures [14].

Alternative non-contact approaches include radar-based and vision-based sensing systems. A comprehensive review by Wu Z. et al. highlighted the capabilities of radar and computer vision techniques for water-level measurement, noting that their performance depends strongly on environmental conditions and installation geometry [15]. In addition, Catsamas S. and colleagues developed a low-cost radar-based IoT sensor, demonstrating the feasibility of non-contact measurements, although such approaches are primarily validated in open-channel environments rather than deep tubular wells [16]. The general limitations of echo-based non-contact methods in confined geometries are further discussed by Shenoy et al., who reviewed the full spectrum of liquid level measurement technologies and concluded that time-of-flight techniques based on direct acoustic propagation offer distinct advantages over reflection-based approaches when the measurement channel introduces multipath ambiguity [17].

In parallel, the integration of sensing systems with Internet of Things (IoT) technologies has enabled distributed monitoring architectures with real-time data transmission. Espinoza Ortiz et al. developed a low-cost IoT-based groundwater monitoring system, illustrating practical implementations of remote sensing and telemetry [18,19]. Furthermore, studies by Alghamdi AM and co-authors investigated LoRaWAN-based communication systems, demonstrating their suitability for long-range, low-power environmental monitoring applications [20]. Additional work by Rajabov F. and Jamolov K. explored IoT integration and cloud connectivity for groundwater monitoring systems, further emphasizing the importance of scalable communication frameworks [21]. Thomas et al. developed a fully open-source, low-cost wireless sensor network for real-time scalable groundwater monitoring, demonstrating that automated telemetry systems can match the data quality of commercial installations at a fraction of the cost, with relevance for distributed aquifer management [22]. Kombo et al. designed and deployed a LoRa-GSM IoT system for monitoring groundwater resources with integrated energy harvesting, validating the feasibility of autonomous, multi-year operation in remote locations without grid power access [23].

From a signal processing perspective, ultrasonic time-of-flight (ToF) measurement has been widely studied to improve accuracy and robustness. Medina C. et al. demonstrated that RF-assisted synchronization can enable highly accurate ultrasonic positioning, while Liu Q. et al. proposed digital lock-in filtering techniques to enhance ToF estimation under noisy and multipath conditions [24,25]. Tanaka et al. provided a detailed energy breakdown for remote IoT sensor nodes, confirming that aggressive duty cycling combined with race-to-sleep firmware strategies and carefully designed power domains are essential to achieving multi-year battery autonomy in unattended field deployments [26].

The specific challenge of power-domain management in duty-cycled embedded systems is addressed by Magno et al., who demonstrated that hierarchical power-switching architectures in which high-consumption analog subsystems are fully disabled between measurement cycles can reduce average current consumption by an order of magnitude while preserving measurement integrity [27]. In the context of LoRa-based water monitoring networks, Chen et al. validated a distributed IoT water environment monitoring system using an STM32L series microcontroller and LoRa communication, confirming that this hardware combination provides an effective and energy-efficient platform for field-deployed sensing nodes, including those requiring periodic activation of power-hungry analog frontends [28]. The importance of packet-level data validation in duty-cycled IoT systems including cyclic redundancy checking and timeout-based rejection of missing responses is demonstrated by Alghamdi et al. in the context of LoRaWAN-based water monitoring, where transient link outages and occasional packet loss were shown to be manageable through controlled retransmission strategies without significant impact on long-term data completeness [29].

Despite these advances, existing approaches remain limited in deep and narrow observation wells, where echo-based ultrasonic systems suffer from multipath ambiguity and reduced effective range, while radar and vision-based systems face practical constraints related to installation and cost. Moreover, many IoT-based monitoring platforms focus on communication and system integration without addressing the fundamental limitations of measurement physics in confined geometries. The confined acoustic propagation environment of a narrow tubular well has received limited dedicated study in the water monitoring literature, though analogous behavior in which tubular geometry losses acts as a quasi-waveguide that reduces lateral energy and supports longer-range propagation than open-air configurations is well established in borehole acoustics and pipe inspection research [30,31]. This physical characteristic has not previously been exploited for groundwater level sensing using a direct one-way propagation paradigm.

The approach proposed in this study addresses this gap by introducing a dual-signal measurement framework that combines RF-based synchronization with direct one-way ultrasonic ToF to a floating receiver. Unlike conventional methods, the proposed system eliminates reliance on echo detection and leverages tubular well geometry to enable long-range and robust measurement. The concept builds upon earlier developments by Rajabov F., including a patented non-contact ultrasonic measurement method and prior prototype systems, and extends these ideas into a fully integrated, low-power, and field-validated monitoring solution [30,31].

## 3. Materials and Methods

### 3.1. Dual-Signal Direct ToF Principle

**Figure 1:**
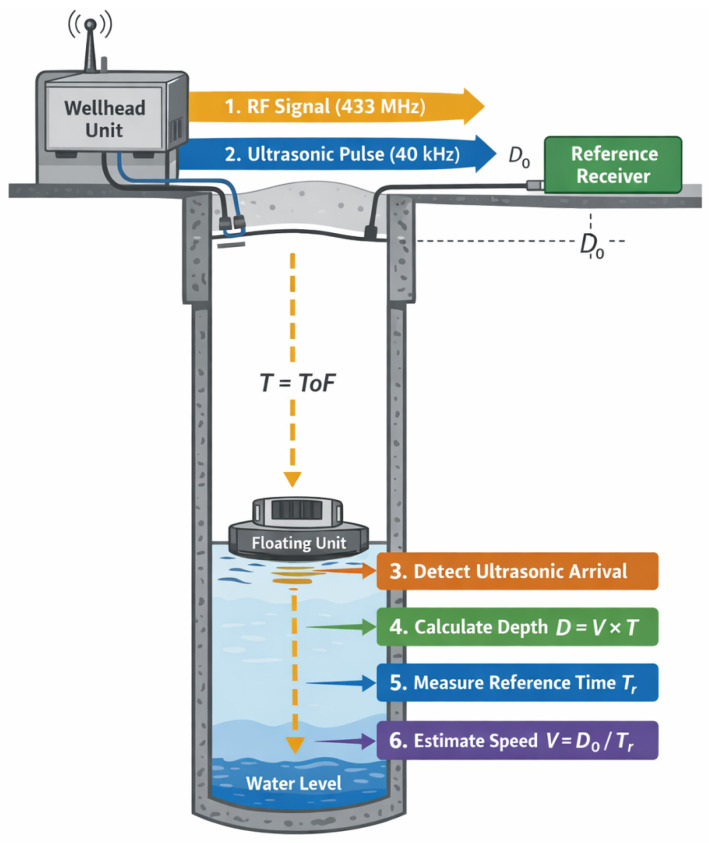
The principle of dual-signal direct ToF measurement.

The proposed method determines groundwater level in an observation well by measuring the one-way airborne ultrasonic time-of-flight (ToF) between a transmitter installed at the wellhead and a receiver positioned on a floating platform at the water surface, as shown Figure 1. The key idea is to estimate the depth from the direct propagation time of the ultrasonic wave rather than from a reflected echo. This operating principle is particularly suitable for deep and narrow observation wells, where conventional echo-based ultrasonic measurement often becomes unreliable because of multipath reflections from the casing wall, fittings, and other structural elements.

In conventional non-contact ultrasonic level measurement, the transducer is usually mounted above the water surface, and the distance is estimated from the time required for the emitted pulse to travel to the surface and return as an echo. In narrow tubular wells, however, the reflected signal may be distorted by repeated reflections, attenuation, and interference, making correct echo identification difficult. To avoid this limitation, the present study adopts a direct one-way propagation scheme. In this configuration, the receiver is placed directly at the water surface, so the measured propagation time corresponds to the actual travel time of the ultrasonic signal from the wellhead to the floating unit. The measurement cycle operates in a sequence of synchronized steps.

**Step 1. Transmission of two synchronized signals:** At the beginning of each measurement cycle, the surface unit simultaneously emits two signals:

1. 433 MHz ISM-band radio signal.
2. 40 kHz ultrasonic burst.

These two signals are generated at nearly the same instant by the wellhead unit. The radio signal is used only for synchronization, whereas the ultrasonic signal is used for physical distance measurement.

**Step 2. Establishment of time-zero reference:** Because electromagnetic waves propagate much faster than acoustic waves, the travel time of the 433 MHz radio signal over the depth of a well is negligible compared with the travel time of the ultrasonic wave through air. Therefore, when the floating unit receives the radio packet, that instant is treated as an effective time-zero reference for the measurement process. In other words, the radio arrival starts the timing operation at the floating receiver.

**Step 3. Detection of ultrasonic arrival:** After the radio reference is received, the floating unit waits for the incoming ultrasonic burst. When the ultrasonic signal is detected by the receiver, the ToF counter is stopped. The elapsed time between the RF synchronization event and the ultrasonic detection event is taken as the one-way ultrasonic propagation time, denoted by T.

**Step 4. Conversion of propagation time to depth:** If the effective sound speed in the air column is known, the groundwater depth can be computed directly from the measured ToF. The basic relationship is:

*D* = *V* × *T* (1)

where *D* is the depth from the transmitter to the floating receiver, *V* is the effective speed of sound, and T is the measured one-way ultrasonic propagation time. Because the estimated depth is directly proportional to the effective sound speed, any uncompensated error in V produces a proportional depth error. Therefore, sound-speed calibration becomes increasingly important as the propagation distance increases.

A critical practical issue is that the speed of sound is not perfectly constant. It varies with temperature, humidity, air composition, and, to a lesser extent, pressure conditions inside the well. If a fixed nominal sound speed is assumed, the depth estimate may contain systematic error, especially in long-range measurements. For this reason, the proposed system includes an optional reference-based calibration mechanism.

**Step 5. Optional reference-channel calibration and its limitation:** To reduce systematic error caused by variations in the speed of sound, an optional reference receiver may be installed at a known and mechanically stable distance *D*_0_ from the transmitter. This reference channel measures the one-way ultrasonic propagation time *T_r_* over the known local path. The effective sound speed along this reference path can then be estimated as

*V* = *D*_0_/*T_r_* (2)

where *V* denotes the locally estimated effective sound speed. This value can be used as an approximate calibration value in the depth calculation when the air column inside the well is assumed to be sufficiently homogeneous.

In the 12-month field validation reported in this study, the optional reference channel was not independently evaluated as a separate correction module. The field results were obtained using an effective calibrated sound-speed value, while the reference-channel concept was retained as an optional calibration mechanism for future deployments. Therefore, the reference-channel estimate should be interpreted as a local correction aid rather than as a guaranteed full-path compensation method.

The reference-channel estimate does not guarantee complete compensation for environmental variations along the entire propagation path between the wellhead transmitter and the floating receiver. In deep wells, vertical gradients of temperature, humidity, airflow, and gas composition may occur. Consequently, the sound speed estimated over the reference path may differ from the path-averaged sound speed over the full transmitter–floating receiver path. A dedicated evaluation of the reference-channel benefit requires additional controlled experiments comparing fixed sound-speed estimation, single reference-channel calibration, and distributed environmental compensation along the well column.

**Step 6. Final depth estimation.** Once V has been estimated from the reference channel, or assigned from a previously validated calibration condition, the floating-unit ToF measurement *T* is converted into the final depth value *D*. This depth represents the distance from the wellhead transmitter to the water surface where the floating receiver is located.

The full measurement principle can therefore be summarized as follows:

- The surface unit emits an RF synchronization signal and an ultrasonic burst at the same time.
- The floating unit receives the RF signal and starts with timing.
- The floating unit receives an ultrasonic signal and stops timing.
- The system calculates the one-way ToF.
- The effective sound speed is estimated using the optional reference channel.
- The groundwater depth is computed from the product of sound speed and measured ToF.

In this study, *D* and *D*_0_ are expressed in meters, *T* and *T_r_* in seconds, and V in meters per second. The reference distance D_0_ does not have to be a fixed standard length; however, it must be selected according to the installation geometry and must remain mechanically stable during operation to preserve calibration accuracy.

This dual-signal direct-ToF principle offers several practical advantages for groundwater monitoring. First, it avoids the ambiguity associated with echo identification in narrow wells. Second, it enables long-range measurements using a physically simple synchronization scheme. Third, it supports adaptation to changing environmental conditions through reference-based sound-speed estimation. As a result, the method is well suited for deep observation wells where conventional echo-based ultrasonic methods are difficult to apply reliably; however, the benefit of reference-based sound-speed correction requires further controlled validation.

### 3.2. System Architecture and Hardware Composition

The proposed groundwater monitoring system consists of three principal subsystems (see Table 1): a surface (wellhead) unit responsible for measurement scheduling, signal generation, and data management; a floating unit located at the water surface for synchronized signal reception and time-of-flight (ToF) estimation; and an optional reference receiver for in situ estimation of the effective sound speed.

The prototype was implemented using commercially available embedded, RF, and ultrasonic components. The surface unit consisted of a Raspberry Pi-class controller, a 433 MHz ISM-band RF/LoRa module, a 40 kHz ultrasonic transmitter, a 12 V ultrasonic excitation stage, regulated logic power rails, and a wellhead mounting structure. The floating unit consisted of an STM32F103C8T6 microcontroller, a 40 kHz ultrasonic receiving element, an analog amplification and filtering front-end, an RF receiving/communication module, a protected power-switching circuit, and a sealed protective enclosure. The receiver front-end was adapted from commercially available waterproof ultrasonic modules, while the onboard control logic was bypassed to allow custom RF-synchronized ToF capture.

The floating unit was positioned directly at the groundwater surface. The ultrasonic receiver was oriented toward the wellhead transmitter to detect the direct incoming acoustic burst. A tethering mechanism was used to limit lateral displacement and to allow retrieval during maintenance. The tether was not used for electrical measurement or distance estimation. Its role was mechanical stabilization and recovery of the floating unit. During maintenance visits, the enclosure, tether condition, receiver orientation, and possible contamination of the receiver surface were inspected.

**Figure 2:**
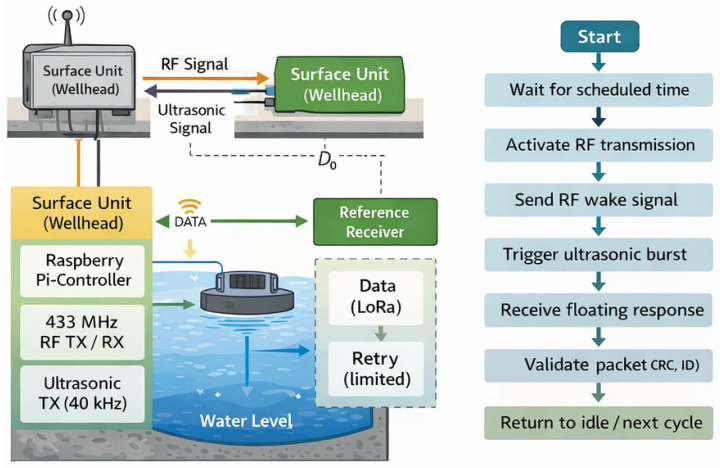
System architecture of the proposed groundwater-monitoring system.

Figure 2 illustrates the overall system architecture, showing the interaction between the surface unit, floating unit, and reference receiver. The separation of the wellhead control unit and the floating receiver reduces dependence on submerged pressure-sensing elements, while keeping the main control, transmission, and data-management electronics at the wellhead.

From an operational perspective, the system follows a structured measurement workflow. The surface unit initiates a synchronized transmission sequence consisting of an RF wake/synchronization signal followed by a timing marker and a 40 kHz ultrasonic burst. The floating unit, which operates in a duty-cycled listening mode, detects the RF signal, activates its receiver circuitry, and performs repeated ToF measurements. The processed result is then transmitted back to the surface unit using the same ISM/LoRa communication link.

To ensure reliable operation under real-world conditions, several fault-tolerance mechanisms are incorporated. These include:

- Multi-shot acquisition to suppress transient disturbances and false detections.
- Packet-level validation using identifiers and cyclic redundancy checks (CRC).
- Time-out-based rejection of missing or invalid responses.
- Controlled retransmission with a limited retry count.

These mechanisms ensure that only valid measurements contribute to the final depth estimation. A limitation of the present prototype description is that some mechanical dimensions and installation details may require adaptation for wells with different diameters, casing materials, and groundwater-surface conditions. Therefore, future work will include standardized mechanical drawings, expanded bill-of-materials-style documentation, and validation in multiple wells with different geometries. This will further improve independent reproducibility and allow more complete assessment of the measurement feasibility under different field conditions.

**Figure 3:**
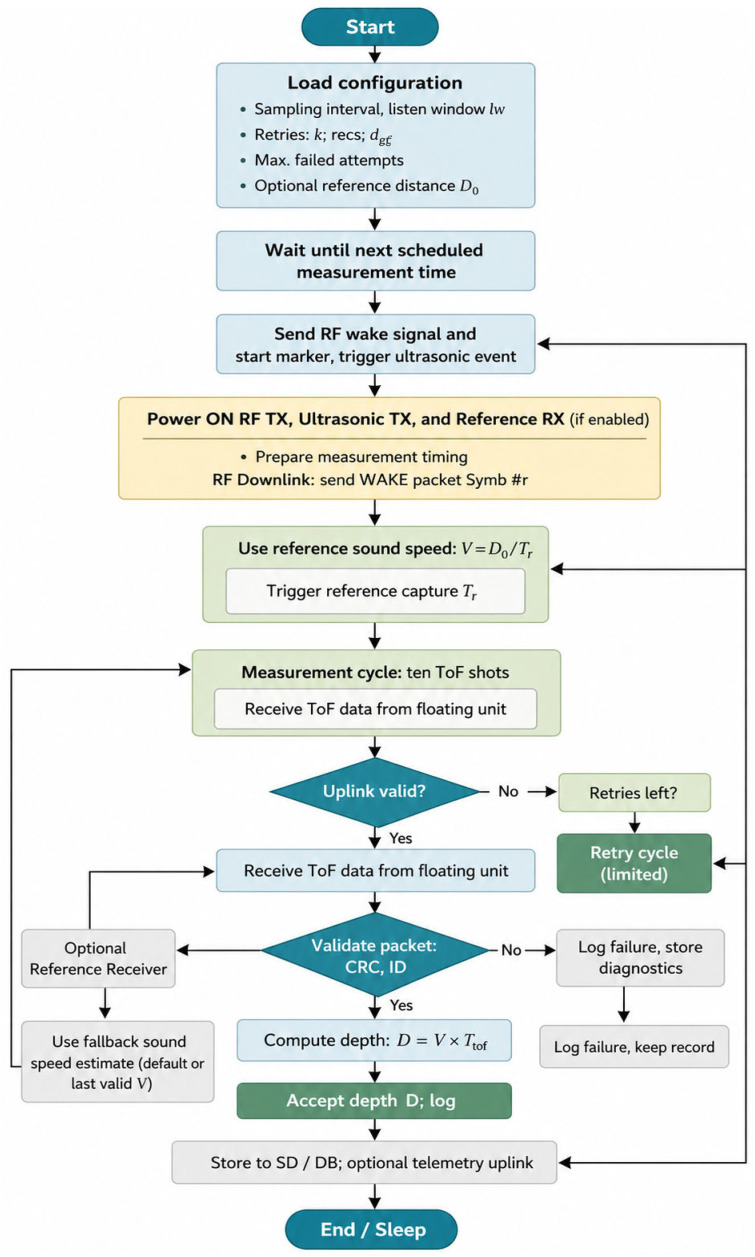
Control algorithm executed by the surface unit during synchronized ToF measurement.

Figure 3 presents the control algorithm executed by the surface unit. The algorithm aligns transmission timing with the floating unit’s listening windows, manages synchronization signaling, and controls measurement retries. This coordination is essential for maintaining reliable operation under aggressive duty-cycled conditions and limited power availability.

**Figure 4:**
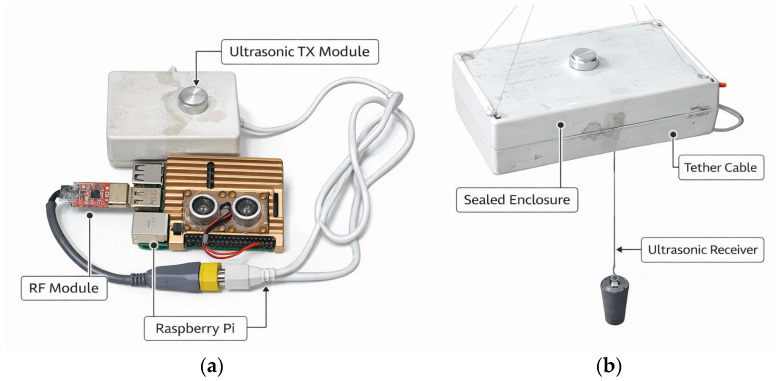
Hardware implementation of the prototype: (**a**) surface (wellhead) unit; (**b**) floating water-surface unit.

Figure 4 presents the hardware implementation of the proposed system, including the wellhead surface unit and the floating unit positioned at the water surface. The surface unit is responsible for measurement scheduling, RF-based synchronization, ultrasonic transmission, and optional reference measurements, while the floating unit performs synchronized ToF acquisition, robust local estimation, and uplink data transmission. This separation of the wellhead control unit and the floating receiver reduces dependence on submerged pressure-sensing components, while keeping the main control, transmission, and data-management electronics at the wellhead. The system is designed using modular architecture, which facilitates reliable operation, ease of deployment, and resilience to environmental conditions.

As shown in Figure 4a, the surface unit integrates the main control and communication components, including the processing board (Raspberry Pi-class controller), RF communication module, ultrasonic transmission module, and power supply interface. These components collectively enable measurement scheduling, synchronization signaling, and generation of high-energy ultrasonic pulses. The compact configuration of the unit allows convenient installation at the wellhead and supports stable operation in field environments. Figure 4b illustrates the floating unit, which is enclosed within a sealed protective housing designed for operation in humid and water-exposed conditions. The enclosure provides protection against moisture, dust, and mechanical impacts. A tethering mechanism ensures positional stability of the floating platform during operation. The ultrasonic receiver is positioned below the floating body to enable direct detection of the propagated acoustic signal. The internal electronics perform synchronized signal reception, ToF measurement, and preliminary data processing before transmitting results to the surface unit. Together, these hardware modules form a RF-synchronized one-way ultrasonic ToF measurement system with a floating receiver, designed for autonomous groundwater monitoring with reduced dependence on submerged pressure-sensing elements. It should be noted that the proposed system is not fully non-contact in the strict physical sense. The measurement principle avoids submerged pressure sensing and echo-based ultrasonic ranging, but it uses a floating receiver positioned at the groundwater surface. Therefore, the floating unit remains exposed to the internal well environment, including humid air, condensation, splashing, possible biofouling, sediment contact, and chemical effects. In the present prototype, the floating unit was enclosed in sealed protective housing and mechanically tethered to maintain stable positioning during operation. Thus, the practical distinction of the proposed architecture is that it reduces dependence on continuously submerged pressure-sensing elements, rather than eliminating environmental exposure.

The proposed architecture provides a robust and scalable solution for groundwater monitoring in deep and remote observation wells. By combining RF-synchronized one-way ToF sensing, low-power embedded operation, and adaptive sound-speed calibration, the system improves measurement stability while reducing dependence on submerged pressure-sensing elements.

### 3.3. Test Groundwater Well, Field Installation, and Mechanical Stabilization

To verify the proposed dual-signal direct time-of-flight (ToF) groundwater monitoring system under realistic operating conditions, field experiments were conducted in a vertical cased observation groundwater well located in the foothill zone of the Tashkent region, Uzbekistan. The site was selected because it represents a typical groundwater-monitoring environment where autonomous long-term water-level observation is required under limited infrastructure conditions. Since the proposed method relies on direct one-way ultrasonic propagation through the air column inside the well, the geometry, casing properties, installation stability, and floating-unit positioning are important factors influencing acoustic propagation, received signal strength, ToF stability, and measurement repeatability. The main characteristics of the test well and installation configuration are summarized in Table 2.

Since casing diameter affects acoustic propagation and floating-unit alignment, the reported installation parameters should be considered part of the experimental configuration rather than universal design constants. Future deployments in wells with different diameters will require geometry-dependent adjustment of receiver positioning, tethering, and detection thresholds.

The surface unit was installed at the wellhead and mechanically fixed to maintain stable orientation during the monitoring campaign. It included the central controller, RF synchronization and communication module, ultrasonic transmitter, ultrasonic excitation circuit, power-supply subsystem, and data-logging interface. A Raspberry Pi-class controller was used for measurement scheduling, control logic, and data recording. RF synchronization and communication were implemented at 433 MHz, while the acoustic ranging signal was generated using a 40 kHz ultrasonic transmitter. The ultrasonic transmitter was oriented downward and aligned approximately along the vertical axis of the well causing direct acoustic propagation toward the floating receiver. The floating unit was deployed directly at the groundwater surface inside the well. It served as the synchronized receiving subsystem and contained the RF receiver/communication module, 40 kHz ultrasonic receiving element, analog front-end, STM32F103C8T6 microcontroller, power-management circuit, and sealed protective enclosure. The ultrasonic receiver was oriented toward the wellhead transmitter to detect the direct incoming acoustic burst. The floating unit was not used as a submerged pressure sensor; instead, it functioned as a water-surface receiver for RF-synchronized one-way ultrasonic ToF measurement.

**Figure 5:**
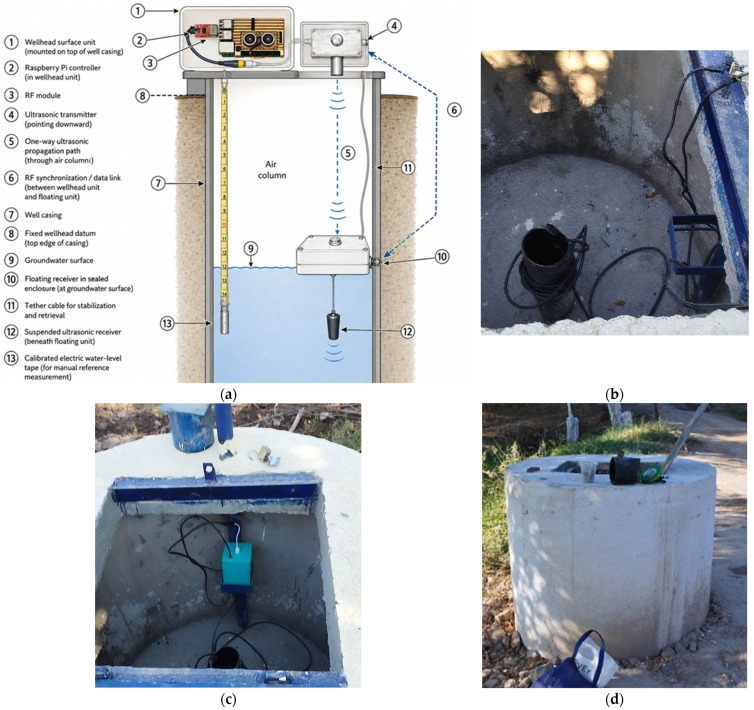
The observation-well test site and installation: (**a**) field installation layout of the proposed groundwater-monitoring system; (**b**) observation-well head and access structure; (**c**) internal wellhead installation with the sensing device and wiring; (**d**) external view of the observation well used for field validation and range-dependent acoustic-link testing. The range-dependent test was conducted inside the internal 170 mm plastic pipeline.

To improve the clarity of the field-deployment configuration, Figure 5 illustrates the actual installation concept used in the field experiment. The figure shows the wellhead-mounted surface unit, the downward-oriented ultrasonic transmitter, the RF synchronization/data link, the floating receiver positioned at the groundwater surface, the tethering arrangement used for mechanical stabilization, and the electric water-level tape used for manual reference measurement.

As shown in Figure 5, the surface unit was mounted at the wellhead and oriented approximately along the vertical axis of the well. The floating receiver was positioned at the groundwater surface and mechanically stabilized using a non-conductive mechanical tether line. During validation visits, the groundwater depth was also measured manually using a calibrated electric water-level tape referenced to the same fixed wellhead datum. This figure helps clarify the physical measurement geometry and the relationship between the automated system and the manual reference procedure.

The floating receiver was designed to follow vertical groundwater-level changes while maintaining approximate axial alignment with the wellhead transmitter. Since the unit floats at the water surface, vertical displacement caused by groundwater-level variation does not require a fixed submerged installation. To reduce uncontrolled lateral drift, the floating enclosure was connected to a non-conductive mechanical tether line. The tether allowed vertical movement with the water surface while limiting horizontal displacement and enabling retrieval during maintenance. During scheduled maintenance visits, the floating unit was inspected for enclosure integrity, tether condition, receiver orientation, receiver-surface contamination, and approximate axial positioning. If necessary, tether tension and receiver orientation were adjusted to restore stable positioning.

The tether used in the present prototype was a passive mechanical tether line rather than an electrical data or power cable. Its function was limited to reducing lateral drift of the floating receiver and enabling retrieval during maintenance. It was not used for signal transmission, electrical measurement, timing synchronization, power delivery, or depth estimation. This distinction is important because using the tether as a communication cable would introduce additional mechanical and electrical constraints. During groundwater-level variation, a wired tether would be repeatedly bent, tensioned, and displaced together with the floating receiver, increasing the risk of fatigue, connector leakage, signal noise coupling, and installation complexity. Therefore, the present design intentionally separates mechanical stabilization from timing and data communication.

The RF link remains necessary in the proposed architecture for two reasons. First, it provides the near-instantaneous timing reference required to start the one-way ToF measurement at the floating receiver. Second, it enables bidirectional data exchange without requiring conductive wiring through the moving floating assembly. Thus, the passive tether is a mechanical stabilization component, whereas the RF module is an active synchronization and communication component. The innovation of the proposed system lies not in wireless telemetry alone, but in the combined architecture of RF-based time-zero synchronization, one-way ultrasonic ToF propagation to a floating receiver, and robust duty-cycled local estimation. In each measurement cycle, the surface unit emitted the RF synchronization signal and the 40 kHz ultrasonic burst. The floating unit detected the RF signal, established the timing reference, received the ultrasonic signal, and measured the one-way ToF. For each cycle, ten ToF samples were acquired and processed using the median-like estimator described in Section 3.5. This repeated-acquisition strategy was adopted to reduce the influence of receiver start-up transients, RF timing jitter, weak ultrasonic arrivals, and occasional false detections.

Independent field validation was performed using a calibrated electric water-level tape, which served as the reference instrument during site visits. The manual reference depth and the proposed system estimate were measured from the same wellhead datum. Over the 12-month campaign, a total of 360 measurement cycles were scheduled, of which 358 cycles were successfully completed and classified as valid, corresponding to a valid detection rate of 99.4%. Across the validation period, the proposed system achieved a mean absolute error (MAE) of 0.048 m and a maximum absolute error (MaxAE) of 0.050 m. These results indicate that the proposed configuration is operationally feasible for groundwater-level monitoring with reduced dependence on submerged pressure sensing during the 12-month field-validation period.

Although the tethering mechanism reduced lateral displacement, it did not eliminate possible movement caused by water-surface disturbance, airflow, mechanical vibration, or changes in well conditions. Therefore, floating-unit stabilization is considered an important installation condition of the present prototype. Future versions of the system will include a standardized centralizing frame or guide structure to improve repeatability in wells with different diameters, casing materials, and water-surface conditions.

### 3.4. Twelve-Month Field Validation Protocol

A 12-month field-validation campaign was conducted to assess the proposed system in terms of depth-estimation accuracy, valid detection rate, and operational stability under long-term duty-cycled deployment. The monitoring period covered seasonal variations in groundwater conditions and environmental exposure, thereby enabling evaluation of the proposed method under changing field conditions rather than under a single short-term test scenario. Each validation session consisted of repeated scheduled measurement cycles executed by the proposed monitoring system, followed by an independent manual reference measurement using a calibrated electric water-level tape. The tape had a scale resolution of 1 mm, while the practical field reading accuracy was approximately ±5 mm, considering contact detection, tape positioning, operator reading, and wellhead datum alignment. Within each measurement cycle, the floating unit first received the RF synchronization signal, then detected the arrival of the one-way ultrasonic pulse, and finally estimated the propagation time after local robust processing. As described in Section 3.3, ten individual ToF measurements were acquired in each cycle. These ten samples were sorted in non-decreasing order, and the final cycle-level ToF estimate was computed as the average of the fifth and sixth ordered samples. This median-like estimator was adopted to reduce the effect of occasional outliers caused by analog front-end settling, RF timing jitter, and false threshold triggering immediately after wake-up.

For each validation date, the proposed depth estimate was compared with the manually measured reference depth. Let $D_{ref}$ denote the groundwater depth measured by the electric water-level tape and $D_{est}$ denote the depth estimated by the proposed system. The absolute error for a given validation instance was defined as

(3) $$AE=|D_{est}-D_{ref}|.$$

Across all valid measurements, the mean absolute error (MAE) was calculated as

(4) $$MAE=\frac{1}{N}\sum_{i=1}^{N}|D_{est,i}-D_{ref,i}|,$$

where N is the number of valid observations. The maximum absolute error (MaxAE) was also reported to characterize the worst observed deviation:

(5) $$MaxAE=\underset{1\leq i\leq N}{max}|D_{est,i}-D_{ref,i}|.$$

The reliability of system operation was quantified using the valid detection rate:

(6) $$R_{valid}(\%)=\frac{N_{valid}}{N_{total}}\times 100,$$

where $N_{valid}$ is the number of cycles that successfully completed RF synchronization, ultrasonic detection, local estimation, and error-free data return, and $N_{total}$ is the total number of attempted cycles. Cycles affected by missing synchronization, absent ultrasonic arrival, timeout events, or invalid communication packets were excluded from the accuracy calculation and counted only in the reliability analysis.

During the 12-month field-validation campaign, depth estimation was performed using an effective calibrated sound-speed value. The optional reference channel was not separately evaluated as an independent correction mechanism during this campaign; therefore, its specific contribution to the reported MAE and MaxAE was not isolated. The validation protocol included two complementary evaluations: routine field validation against manual electric-tape reference measurements and a separate range-dependent test for assessing valid ToF detection under confined tubular propagation conditions. The detailed range-dependent results are presented in Section 4.5.

### 3.5. Power Architecture, Distribution Switching, and Duty-Cycled Operation

The system is designed for long-term battery-powered deployment using aggressive duty-cycling strategies. In normal operation, the floating unit remains in deep sleep and activates only during short, scheduled listening windows. Measurement cycles are executed at configurable intervals ranging from one cycle every 10 min to one cycle per day, allowing a trade-off between temporal resolution and energy autonomy.

Power-domain management is implemented through controlled switching of high-consumption subsystems. A protected load switch is used to repeatedly enable and disable power to the ultrasonic receiver front-end and communication modules. A dual-channel power distribution switch (MIC2026/MIC2076 family) provides current limiting, fault protection, and controlled switching behavior, ensuring reliable operation during frequent power cycling. Both the surface and floating units employ DC–DC conversion to generate stable 3.3 V and 5 V logic supply rails, together with a dedicated 12 V rail required for ultrasonic transmission. This multi-rail architecture enables low standby power consumption while providing sufficient peak power during short acquisition intervals. As a result, the system is suitable for deployment in remote or difficult-to-access monitoring locations.

Synchronization between the surface and floating units is achieved using short RF transmissions that serve both as wake-up signals and as timing references for ToF measurement. During standby, the floating unit activates only its RF receiver within predefined listening windows. Upon detection of a designated synchronization packet, the microcontroller exits deep sleep and enables the ultrasonic reception chain. A subsequent timing marker, implemented either as a second RF symbol or as a specific packet field, defines the start of the ToF counter, while detection of the ultrasonic signal defines the stop event. The elapsed time between these two events corresponds to the one-way ultrasonic propagation time.

To improve robustness, each measurement cycle includes ten independent ToF acquisitions, denoted as $T_{1},T_{2},\ldots ,T_{10}$. After sorting the acquired samples in non-decreasing order, $T_{1}\leq T_{2}\leq \ldots \leq T_{10},$ the final ToF estimate is computed using the median-like estimator:

(7) $$\hat{T}=\frac{T_{5}+T_{6}}{2}.$$

This estimator suppresses outliers caused by receiver startup transients, RF synchronization jitter, and occasional false ultrasonic detections, while remaining computationally efficient for embedded implementation. The estimated groundwater depth is then obtained as

(8) $$D=V\times \hat{T},$$

where V is either obtained from the reference channel $\left(V=D_{0}/T_{r}\right)$ or derived from the most recent calibration state.

For long-range deployments, the scheduling mechanism also contributes to measurement reliability. By restricting operation to predefined acquisition windows, the system reduces both energy consumption and the likelihood of unintended triggers outside the measurement interval. After completing the multi-shot acquisition, estimation, and optional data transmission, the floating unit disables non-essential subsystems via the power distribution switch and returns to deep sleep, restoring the system to its lowest power state until the next scheduled measurement cycle.

## 4. Results

### 4.1. Range-Dependent Confined-Tubular Acoustic-Link Test

To evaluate the maximum stable acoustic-link capability of the proposed RF-synchronized one-way ultrasonic ToF architecture, a separate range-dependent confined-tubular propagation experiment was conducted. This experiment was independent of the 12-month groundwater-level field validation described in Section 3.4. The 12-month field validation was performed under actual groundwater-monitoring conditions, where the groundwater depth varied mainly between 14 m and 30 m below the wellhead datum. In contrast, the range-dependent experiment was designed to evaluate whether the transmitter–receiver configuration could maintain valid ToF detection over longer known propagation distances.

The range-dependent experiment was conducted inside a straight plastic pipeline installed in the observation-well structure. The inner diameter of the pipeline was approximately 170 mm. This pipeline provided a confined tubular acoustic path similar to the operating geometry of a narrow-cased observation well. The same 40 kHz ultrasonic transmitter, RF synchronization module, receiver electronics, analog front-end, and ToF detection algorithm were used during this test. The transmitter was fixed near the wellhead side of the pipeline, while the receiver unit was placed at known axial separations inside the pipeline.

The tested axial separations between the transmitter and receiver were 50, 100, 150, 200, 250, 300, 325, and 350 m. These distances were established using a calibrated marked suspension/measuring line referenced to the transmitter datum. For each distance, the receiver unit was lowered or positioned along the plastic pipeline until the required marked distance was reached. The receiver was maintained approximately along the pipeline axis to reproduce the direct one-way propagation geometry of the field system. Therefore, the 50–350 m distances represent controlled transmitter–receiver separations inside the 170 mm plastic pipeline, not natural groundwater depths observed during the 12-month field-validation campaign.

After positioning the receiver at each distance, 30 measurement cycles were performed. A measurement cycle was classified as valid only when all the following conditions were satisfied:

- The RF synchronization packet was correctly received;
- The ultrasonic arrival was detected within the expected time window;
- The measured ToF was physically consistent with the known test distance;
- The returned data packet passed identifier and CRC validation.

The reference value for the error metrics in this experiment was the known transmitter–receiver separation distance measured along the plastic pipeline. Therefore, the range-dependent MAE values were calculated relative to the known test distances, whereas the 12-month field-validation MAE was calculated relative to the calibrated electric water-level tape. For each valid cycle, the estimated distance was calculated as

$${\hat{D}}_{i}=V_{cal}{\hat{T}}_{i},$$

where ${\hat{D}}_{i}$ is the estimated distance for the i-th valid cycle, $V_{cal}$ is the calibrated effective sound speed used during the range-dependent test, and ${\hat{T}}_{i}$ is the measured one-way ToF after local estimation. The absolute error was calculated as

$$AE_{i}=|{\hat{D}}_{i}-D_{ref}|,$$

where $D_{ref}$ is the known axial transmitter–receiver separation.

**Figure 6:**
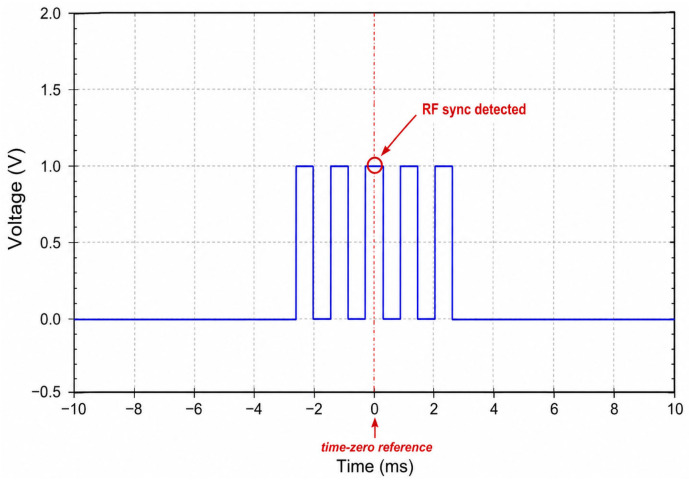
Representative RF synchronization signal used to establish the time-zero reference for one-way ultrasonic ToF measurement.

To make the timing process of the proposed direct-ToF method clearer, representative signal-level examples are provided. Figure 6 shows the RF synchronization signal used to establish time-zero reference at the receiver. The detection point of the RF packet defines the start of the ToF counter before the ultrasonic arrival is measured. Since the propagation time of the RF signal is negligible compared with the ultrasonic propagation time over the tested distances, this RF detection event is used as the timing reference for the subsequent ultrasonic arrival measurement.

**Figure 7:**
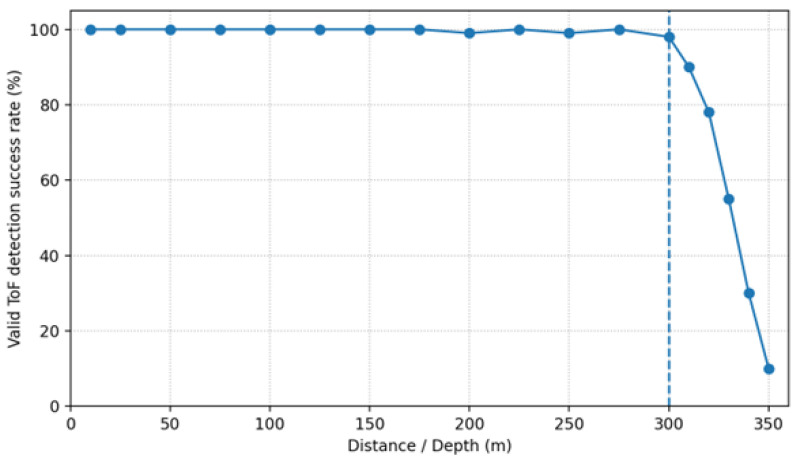
Valid ToF detection rate obtained from the range-dependent confined-tubular acoustic-link test. At each known transmitter–receiver separation, 30 measurement cycles were performed. A cycle was counted as valid only when RF synchronization, ultrasonic arrival detection, ToF consistency checking, and packet validation were completed successfully. The decrease beyond 300 m indicates the transition from stable detection to threshold-limited detection caused by acoustic attenuation, wall interaction, reduced SNR, and increased timing uncertainty.

Figure 7 summarizes the valid ToF detection rate obtained during the range-dependent confined-tubular acoustic-link test. The plotted values were calculated from repeated measurement cycles conducted at the known axial separations of 50, 100, 150, 200, 250, 300, 325, and 350 m. At each distance, 30 measurement cycles were performed.

The behavior of the valid ToF detection rate in Figure 7 can be explained by the distance-dependent received acoustic amplitude and the receiver detection threshold. In open-air free-field propagation, acoustic pressure amplitude approximately follows a 1/r decrease, and acoustic intensity approximately follows a $1/r^{2}$ decrease. Therefore, detecting a 40 kHz ultrasonic burst at several hundred meters would be difficult in an unconfined open-air environment. However, the present range-dependent experiment was conducted under confined tubular propagation conditions. In such a geometry, the tube boundary restricts lateral spreading of the acoustic field, and the propagation path behaves as a lossy duct-like channel rather than an ideal free-field spherical radiator.

The received amplitude can be approximated as

$$A(d)=A_{0}d^{-n}exp(-\alpha_{eff}d),$$

where A(d) is the received acoustic amplitude at distance d, $A_{0}$ is the initial amplitude, n is the effective geometrical spreading exponent, and $\alpha_{eff}$ is the effective attenuation coefficient representing air absorption, wall interaction, scattering, leakage, and imperfect alignment. For ideal free-field propagation, n≈1. For a confined guided path, n can be smaller, although $\alpha_{eff}>0$ still causes distance-dependent attenuation.

The receiver detects an ultrasonic arrival only when the received signal-to-noise ratio exceeds the detection threshold:

$$SNR(d)=\frac{A(d)}{\sigma_{n}}>\gamma ,$$

where $\sigma_{n}$ is the receiver noise level and γ is the detection threshold. At distances up to 300 m, the received burst remained sufficiently above the detection threshold under the tested confined tubular condition, resulting in stable valid detection. Beyond 300 m, the received signal approached the threshold region. In this region, small changes in attenuation, alignment, receiver noise, or waveform distortion increased the probability of missed arrivals and invalid ToF estimates. This explains the sharper decrease in the valid ToF detection rate beyond 300 m.

Table 3 summarizes representative water-level measurement technologies and shows that each approach has different advantages depending on the deployment scenario. Pressure-based sensors, such as the Solinst Levelogger 5 and Keller DCX-22 AA, provide high accuracy and are widely used in groundwater monitoring. However, they require submerged pressure-sensing elements, which may increase maintenance needs during long-term deployment, especially in deep wells. Echo-based ultrasonic systems are generally simple and low-cost, but they rely on reflected acoustic signals. Therefore, their performance can be affected by surface disturbance, multipath reflections, and installation geometry. Radar-based systems can provide high accuracy in suitable open-surface environments, but they were not specifically evaluated for one-way acoustic propagation in narrow cased observation wells.

The proposed RF-synchronized direct-ToF system addresses a different measurement scenario. It uses one-way ultrasonic propagation from the wellhead transmitter to a receiver located at the water-surface position, avoiding echo interpretation in narrow tubular geometries and reducing dependence on submerged pressure-sensing elements. Its reported MAE of 0.048 m and MaxAE of 0.050 m are not better than high-end pressure or radar sensors in absolute accuracy. However, the contribution of the proposed system is its suitability for confined tubular observation-well conditions, stable acoustic-link ToF detection up to 300 m under the tested confined-tubular condition, and compatibility with duty-cycled IoT-based monitoring.

The reported 300 m result should therefore be interpreted as an acoustic-link valid-detection result obtained under a separate controlled confined-tubular test condition. It should not be interpreted as evidence of 12-month groundwater-depth validation over the full 300 m interval.

### 4.2. Accuracy of Depth Estimation

The accuracy of the proposed system was evaluated through monthly field measurements conducted at an observation well located in the Tashkent foothills. Reference depth values were obtained using a calibrated electric water-level tape during each measurement campaign. For each month, the proposed depth estimate was computed as the mean value across N valid measurement cycles, while the within-month variability was quantified using the standard deviation. The monthly mean absolute error was defined as the absolute difference between the estimated depth and the reference measurement. Table 4 summarizes the validation results over a 12-month period. The system achieved a mean absolute error (MAE) of 0.048 m and a maximum absolute error (MaxAE) of 0.050 m, computed across monthly mean values. The overall valid detection rate was 358 out of 360 measurement cycles (99.4%), indicating highly reliable operation under real-world conditions.

The results demonstrate that the combination of RF-based synchronization, direct ultrasonic ToF measurement, and robust multi-shot estimation enables consistent and accurate depth estimation across varying environmental conditions. The achieved accuracy is sufficient for typical groundwater monitoring applications, including seasonal trend analysis, threshold detection, and long-term resource assessment. It should be noted that occasional discrepancies observed in late-year measurements are attributed to environmental variability and measurement conditions; however, these do not significantly affect the overall statistical performance.

To provide a more comprehensive view of the 12-month field campaign, additional statistical summaries were derived from the monthly validation results. Table 5 reports overall agreement metrics between the proposed system and the manual electric water-level tape, including MAE, RMSE, mean bias error, and coefficient of determination.

### 4.3. Effect of Multi-Shot Acquisition and Median-like Filtering

The experimental results confirm that multi-shot acquisition combined with median-like filtering plays a critical role in achieving robust ToF estimation. During system operation, the floating unit transitions from deep sleep to active measurement mode, during which transient effects may occur due to analog front-end stabilization, RF synchronization jitter, and threshold adaptation. Single-shot ToF measurements are therefore susceptible to outliers and occasional timing errors. To mitigate this effect, each measurement cycle includes ten consecutive ToF acquisitions, which are sorted and processed using a median-like estimator defined as the average of the fifth and sixth ordered samples. This approach provides an effective compromise between computational simplicity and statistical robustness. It suppresses extreme values while preserving the central tendency of the measurement distribution, making it suitable for real-time embedded implementation.

To illustrate the behavior of the 10-shot estimator, Table 6 presents representative raw ToF samples acquired during one measurement cycle at a reference depth of approximately 22 m. The samples include small timing variations and one abnormal value caused by transient receiver response. The arithmetic mean is affected by this abnormal value, whereas the median-like estimator remains close to the central cluster of samples.

For this example, the single first-shot estimate is 64.05 ms, the arithmetic mean of all ten samples is 64.124 ms, and the proposed median-like estimate is calculated as (64.07 + 64.08)/2 = 64.075 ms. This example shows that the arithmetic mean is shifted upward by the abnormal sample, while the median-like estimator remains representative of the central ToF cluster.

**Figure 8:**
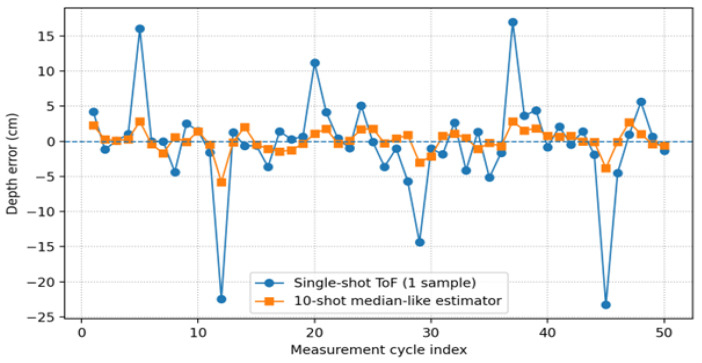
Comparison of single-shot ToF estimation and the 10-shot median-like estimator.

Figure 8 presents a comparison between single-shot ToF measurements and the proposed multi-shot estimator. The single-shot measurements exhibit higher dispersion and occasional outliers, particularly during wake-up transitions. In contrast, the multi-shot estimator significantly reduces variability and produces stable estimates, which directly contributes to the achieved measurement accuracy.

### 4.4. Measurement Interval Configuration and Operational Duty-Cycle

The system supports configurable measurement intervals ranging from one measurement every 10 min to one measurement per day. This flexibility enables adaptation to different monitoring requirements by balancing temporal resolution and energy consumption. To minimize power consumption, the floating unit operates in a duty-cycled mode, remaining in deep sleep outside scheduled measurement windows. During each active window, the unit enables the RF receiver, detects the synchronization signal, performs ToF measurement, and transmits the result before returning to low-power mode.

Table 7 summarizes the available operational modes. Short sampling intervals provide high temporal resolution and are suitable for dynamic conditions such as pumping tests or irrigation periods, but result in higher energy consumption. In contrast, longer intervals substantially reduce the duty cycle and extend battery life, making them more appropriate for long-term monitoring in remote locations. This configurable strategy allows the proposed system to support both high-frequency monitoring and long-term autonomous deployment without hardware modification.

Overall, the field results demonstrate that the proposed system achieves reliable long-range measurements, high valid-detection performance, and stable depth estimation under real observation-well conditions. The combination of direct one-way ToF measurement, RF-based synchronization, robust multi-shot estimation, and duty-cycled operation provides a practical framework for groundwater-level monitoring in deep observation wells.

### 4.5. Extended Experimental Analysis

To complement the 12-month groundwater-level field validation, an extended range-dependent experimental analysis was conducted to evaluate the behavior of the proposed RF-synchronized one-way ultrasonic ToF system under increasing propagation distances. The purpose of this analysis was to clarify the operating range of the developed prototype, quantify the variation in ToF stability with distance, and explain the degradation observed beyond the stable operating region.

It is important to distinguish this extended range-dependent test from the 12-month field validation. The 12-month validation evaluated routine groundwater-depth estimation under actual monitoring conditions, where the groundwater level varied mainly between approximately 14 m and 30 m below the wellhead datum. In contrast, the extended range-dependent test was performed under confined tubular propagation conditions using known transmitter–receiver separation distances. This test was designed to evaluate the stable valid-detection capability of the acoustic ToF architecture at longer propagation distances. The range-dependent test was conducted at representative propagation distances of 50, 100, 150, 200, 250, 300, 325, and 350 m. At each distance, 30 measurement cycles were performed. Each measurement cycle consisted of RF synchronization, 40 kHz ultrasonic burst transmission, ultrasonic arrival detection, ToF estimation, and data-packet validation. A measurement cycle was classified as valid only when the RF synchronization packet was correctly received, the ultrasonic arrival was detected within the predefined time window, the estimated ToF value was physically consistent with the tested distance, and the returned data packet passed identifier and CRC validation. The valid detection rate was calculated as

(9) $$Valid\;detection\;rate\;\left(\%\right)=\frac{N_{valid}}{N_{total}}\times 100$$

where *N_valid_* is the number of valid measurement cycles and *N_total_* is the number of attempted cycles at the corresponding distance.

For each valid cycle, the distance estimate was obtained from the measured one-way ToF and the effective sound speed. The reference value for the error metrics in Table 8 was the known transmitter–receiver separation distance used in the range-dependent test. Therefore, the mean absolute error in Table 8 represents the average absolute difference between the proposed system estimate and the known reference test distance, not the electric-tape field-validation reference.

Table 8 summarizes the range-dependent performance of the proposed direct-ToF system. The results show that the system maintained full valid-detection performance from 50 m to 300 m under the tested confined tubular condition. Over this interval, the mean absolute error increased from 0.012 m at 50 m to 0.048 m at 300 m, while the ToF standard deviation increased from 0.6 ms to 2.6 ms. This indicates that measurement uncertainty increases with propagation distance, mainly because of acoustic attenuation, reduced received signal amplitude, and increased timing uncertainty. Beyond 300 m, a degradation in detection quality was observed. At 325 m, the valid detection rate decreased to 93.3%, and at 350 m it decreased further to 86.7%. The corresponding error values also increased. This behavior is attributed to the reduction in the received ultrasonic signal amplitude at longer distances. As the propagation path becomes longer, acoustic absorption, scattering from casing or tube irregularities, imperfect axial alignment, and receiver threshold uncertainty reduce the signal-to-noise ratio. Consequently, the probability of missed ultrasonic arrivals, false threshold crossings, and invalid ToF estimates increases. Therefore, the 300 m value should be interpreted as the stable valid-detection limit of the current prototype under the tested confined tubular propagation condition. It should not be interpreted as evidence that the proposed system is universally more accurate than all existing water-level monitoring technologies. Commercial pressure transducers and high-end radar sensors may provide higher absolute accuracy under suitable installation conditions. The main contribution of the proposed system is not superior absolute precision or proven lower cost, but its RF-synchronized one-way ultrasonic ToF architecture, which avoids echo-based ranging ambiguity and reduces dependence on submerged pressure-sensing elements in deep observation wells.

For the error metrics reported in Table 8, the reference value was the known test distance between the ultrasonic transmitter and the receiver in the range-dependent confined-tubular experiment. For each valid cycle, the estimated distance was calculated from the measured one-way ToF and the effective sound speed. The absolute error was then computed as the absolute difference between the estimated distance and the corresponding known reference distance. Therefore, the mean absolute error in Table 8 represents the average distance-estimation error over all valid cycles at each tested propagation distance.

The results indicate that the proposed system maintained stable valid ToF detection up to 300 m under the tested confined tubular propagation condition. Beyond 300 m, the valid detection rate decreased and the error values increased. This degradation is attributed to reduced received ultrasonic amplitude, acoustic attenuation, scattering from casing or tubular-wall irregularities, imperfect axial alignment, lower signal-to-noise ratio, and increased timing uncertainty. Therefore, the 300 m result should be interpreted as the stable valid-detection limit of the current prototype under the tested confined tubular condition, rather than as a 12-month groundwater-depth validation over the full 300 m interval.

A second part of the extended analysis evaluated the effect of robust repeated acquisition. Since the floating unit operates under duty-cycled conditions, individual measurements may be affected by RF timing jitter, analog front-end settling, weak ultrasonic arrivals, or occasional false detections. To reduce the influence of these effects, the proposed system uses a 10-shot median-like estimator. In each measurement cycle, ten ToF samples are acquired, sorted, and the final estimate is calculated from the central ordered samples. This strategy reduces sensitivity to outliers while remaining simple enough for embedded implementation. As shown in Table 9, the arithmetic mean improves performance compared with single-shot estimation because it uses repeated measurements. However, it remains sensitive to occasional abnormal ToF values. The proposed median-like estimator provides the best overall performance because it suppresses extreme samples before calculating the final ToF estimate. Compared with single-shot estimation, the median-like estimator reduced MAE from 0.073 m to 0.048 m, RMSE from 0.081 m to 0.048 m, and MaxAE from 0.124 m to 0.050 m. It also reduced the outlier rate from 5.3% to 0.6%. These results confirm that the 10-shot median-like estimator is important for stable ToF estimation under duty-cycled field operation.

Taken together, Table 8 and Table 9 provide additional experimental evidence for the range-dependent behavior and estimator robustness of the proposed system. Table 8 shows that stable valid detection was maintained up to 300 m under the tested confined tubular condition, while detection quality decreased beyond this distance because of reduced acoustic signal strength and increased timing uncertainty. Table 9 demonstrates that the 10-shot median-like estimator substantially improves measurement stability compared with single-shot estimation. These results support the conclusion that the combination of RF-synchronized one-way ToF measurement and robust repeated acquisition is essential for reliable operation of the proposed prototype. However, comparison with existing technologies should be interpreted carefully. The proposed system is not claimed to be more precise than commercial pressure transducers or high-end radar sensors in absolute terms, and no complete cost comparison is presented in this study. Instead, the proposed method addresses a specific measurement scenario: deep observation wells where echo-based ultrasonic ranging can be unreliable because of multipath ambiguity and where reducing dependence on submerged pressure-sensing elements is desirable.

### 4.6. System-Level Component Analysis

To further clarify how the reported field performance arises from the overall system design, Table 10 presents a component-level comparative analysis of the proposed architecture. The purpose of this table is to summarize the relative contribution of the principal design elements, including RF synchronization, direct one-way ultrasonic ToF measurement, robust 10-shot estimation, and duty-cycled operation. In this way, the table provides a structured system-level perspective on why the full proposed configuration achieves stronger performance than reduced or alternative configurations.

As shown in Table 10, the full proposed system achieves the strongest overall performance, with an MAE of 0.048 m, an RMSE of 0.048 m, and a detection rate of 99.4%. When the 10-shot estimator is omitted, both error and variability increase, indicating that repeated acquisition plays a major role in suppressing transient disturbances and outliers. When reference calibration is not used, the detection rate remains high, but the error increases, suggesting that sound-speed calibration mainly reduces systematic bias under environmental variation. Likewise, shortened duty-cycle recovery leads to higher errors and lower detection rates, demonstrating that adequate stabilization time after wake-up is important for reliable operation. Finally, the reflection-based baseline shows the weakest performance in the same well environment, consistent with the stronger multipath sensitivity of echo-based approaches in confined tubular geometries.

Overall, the extended analyses in Table 8, Table 9 and Table 10 show that the reported field-level performance is not produced by a single subsystem in isolation, but by the interaction of several mutually reinforcing design choices. Direct one-way ultrasonic propagation reduces ambiguity associated with reflected echoes, RF synchronization provides an effective timing reference, the 10-shot estimator improves robustness against transient disturbances, and the duty-cycled design enables long-term low-power operation. In this way, the extended analysis strengthens the interpretation of the reported results and clarifies the system-level contribution of the proposed method for groundwater monitoring in deep observation wells.

## 5. Discussion

The validated operating range of up to 300 m indicates that the proposed direct ToF approach benefits from the tubular propagation environment characteristic of observation wells. In such confined geometries, acoustic energy tends to remain aligned along the well axis, reducing lateral dispersion and limiting losses associated with off-axis scattering. Compared to open-air ultrasonic propagation, this quasi-waveguide behavior enables more efficient energy transfer over long distances. In the proposed configuration, the ranging event is defined by a one-way ultrasonic arrival at a floating receiver rather than by a reflected echo. This fundamentally reduces ambiguity caused by multipath reflections from casing walls, joints, and internal structures. The high detection success rate observed within the stable operating region (up to 300 m) is therefore consistent with the physical characteristics of confined acoustic propagation. Beyond this range, the gradual decline in detection probability can be attributed to cumulative attenuation, residual multipath effects, and reduced signal-to-noise ratio at the receiver threshold.

Most existing low-cost ultrasonic water-level monitoring systems employ echo-based ranging, where the transducer is positioned above the water surface and measurements rely on reflected signals. While such systems are effective in open or semi-confined environments, they are inherently sensitive to reflection quality and multipath interference, which limits their applicability in deep, narrow wells. Previous studies on ultrasonic sensing for water-level monitoring have primarily focused on energy efficiency, IoT integration, and remote data reporting, rather than on the constraints imposed by tubular propagation environments. Comparative analyses with pressure-based sensors further emphasize installation convenience and long-term stability, but do not address the fundamental limitations of reflection-based ultrasonic measurement in confined geometries. In contrast, the proposed method introduces a structurally different measurement paradigm. By combining RF-based synchronization (433 MHz ISM band) with direct ultrasonic propagation to a floating receiver, the system eliminates dependence on echo interpretation and is inherently better suited for deep observation wells. From a system perspective, this work complements existing IoT-based monitoring platforms by strengthening the reliability of the sensing layer, which can be integrated into broader data acquisition and communication frameworks.

A key practical outcome of the study is the demonstration that robust estimation plays a central role in achieving field-grade measurement accuracy. The system achieved a mean absolute error of 4.8 cm and a maximum absolute error of 5 cm using the proposed multi-shot acquisition strategy combined with a median-like estimator. The necessity of repeated sampling arises from the duty-cycled operation of the floating unit. After exiting deep sleep, both the ultrasonic receiver front-end and the RF synchronization path may exhibit transient behavior, including analog settling effects and timing jitter. Under such conditions, single-shot ToF measurements are susceptible to occasional outliers. The adopted approach collecting ten consecutive ToF samples, sorting them, and computing the estimate as the average of the fifth and sixth values—provides a computationally efficient mechanism for outlier suppression. This estimator preserves the central tendency of the measurement distribution while rejecting extreme values, making it well suited for implementation on resource-constrained embedded systems. The comparison between single-shot and filtered measurements confirms that this strategy significantly reduces variability and improves repeatability.

Despite the favorable results, several limitations should be considered for practical deployment. First, the long-range performance depends on the well structure acting as a relatively continuous acoustic propagation channel. Significant obstructions, strong airflow within the borehole, or irregular internal geometries may degrade signal integrity and reduce detection probability beyond the stable range. Second, while the reference channel enables in situ estimation of the effective sound speed, its accuracy depends on the mechanical stability of the reference distance D_0_ and the consistent placement of the reference receiver. Therefore, D_0_ should be selected to ensure long-term stability under expected environmental conditions. Third, although the use of a protected power-distribution switch (MIC2026/MIC2076) ensures reliable power cycling and fault protection, the overall system lifetime remains dependent on duty-cycle configuration and power conversion efficiency. Careful selection of sampling intervals is therefore required to balance measurement resolution and energy consumption.

### Sound-Speed Sensitivity and Environmental Effects

The proposed direct-ToF method depends directly on the effective speed of sound in the well air column. Therefore, temperature, humidity, pressure, airflow, and vertical air stratification can influence the estimated groundwater depth. The basic distance equation is

(10) D=V×T

where *D* is the estimated distance, *V* is the effective sound speed, and *T* is the measured one-way ultrasonic ToF. If the sound speed used in the calculation differs from the actual path-averaged sound speed by Δ*V*, the corresponding distance error can be approximated as

(11) ΔD ≈ T×ΔV

This relationship shows that sound-speed uncertainty produces a distance-proportional error. Assuming a nominal sound speed of approximately 343 m/s, the expected uncompensated depth error for several representative sound-speed deviations is summarized in Table 11.

Table 11 shows that sound-speed uncertainty is relatively limited for shallow field depths but becomes significant at long propagation distances. For example, an uncompensated sound-speed error of 0.6 m/s, which is approximately equivalent to a 1 °C temperature error in dry air, may produce an error of about 0.039 m at 22 m, but about 0.525 m at 300 m. Therefore, long-range use of the proposed method requires careful calibration of the effective sound speed or additional environmental compensation.

Temperature is the dominant factor affecting the speed of sound in air. Around ordinary environmental conditions, the speed of sound changes by approximately 0.6 m/s per 1 °C. Humidity also affects sound speed, although its magnitude depends on temperature and relative humidity. Pressure has a smaller direct influence under ordinary atmospheric conditions because the speed of sound in an ideal gas mainly depends on temperature and gas composition. However, in deep wells, air stratification, airflow, and vertical temperature gradients may cause the path-averaged sound speed between the transmitter and the floating receiver to differ from the value measured near the wellhead or along a short reference path. For this reason, the reported accuracy should be interpreted together with the sound-speed calibration condition. During short propagation distances, small sound-speed deviations produce limited depth error. At extended distances, however, the error becomes proportional to distance and may dominate measurement uncertainty. Future deployments will therefore include temperature/humidity sensing, periodic sound-speed calibration, and controlled comparison of fixed sound-speed estimation, single reference-channel calibration, and multi-point environmental compensation.

The applicability of the proposed direct-ToF method also depends on well geometry and internal well conditions. In straight and relatively narrow cased wells, the casing can partially guide acoustic energy along the vertical axis, supporting stable one-way ultrasonic propagation. However, the same behavior cannot be assumed for all wells. In larger-diameter wells, acoustic energy may spread more laterally, and the floating receiver may drift farther from the axial propagation path, reducing received signal amplitude and valid detection probability. Casing material, wall thickness, internal roughness, and joints can also affect attenuation, scattering, and multipath behavior. In wells containing bends, pumps, cables, obstacles, or irregular internal structures, the direct acoustic path may be partially blocked or distorted, leading to missed detections or unstable ToF estimates. Strong airflow or ventilation inside the well may further disturb the acoustic path and alter the effective speed of sound. Therefore, the reported performance should be interpreted as valid for the tested straight cased-well configuration and not as a universal result for all well geometries. Future deployments in wells with different diameters, casing materials, bends, obstacles, or strong airflow will require geometry-specific calibration, receiver centralization, adjustment of detection thresholds, and additional field validation.

The mechanical tether should not be interpreted as a replacement for the RF synchronization and data link. In the present prototype, the tether is electrically passive and serves only for stabilization and retrieval. Although a wired communication cable could theoretically be used, it would introduce additional sealing, fatigue, installation, and noise-coupling issues because the floating unit moves with the groundwater surface. The RF link avoids these constraints while also providing the time-zero synchronization needed for one-way ToF measurement. Therefore, the tether and RF link serve different functions and are not redundant components.

Finally, monthly aggregation of measurements was adopted in this study to align with standard groundwater monitoring practices, where short-term fluctuations such as those caused by pumping are suppressed in favor of seasonal and long-term trends relevant to water resource management. Overall, the results and analysis demonstrate that the proposed system provides a reliable and practical solution for long-range groundwater level monitoring in deep observation wells, while maintaining compatibility with low-power operation and scalable monitoring frameworks.

## 6. Conclusions

This study presented a dual-signal direct time-of-flight (ToF) method for groundwater-level monitoring in deep observation wells. The proposed approach combines RF-based synchronization with one-way airborne ultrasonic propagation from a wellhead transmitter to a floating receiver located at the groundwater surface. Unlike conventional echo-based ultrasonic ranging, the method does not rely on detecting a reflected signal from the water surface. Therefore, it reduces echo ambiguity and multipath-related uncertainty in narrow tubular well geometries. The developed prototype integrates a wellhead surface unit, a floating receiver unit, duty-cycled power operation, and a multi-shot ToF acquisition strategy with a median-like estimator. The field validation was conducted over a 12-month period under actual groundwater-monitoring conditions, during which the groundwater depth varied between approximately 14 m and 30 m below the wellhead datum. Within this field-validation interval, the proposed system achieved a mean absolute error of 0.048 m, a maximum absolute error of 0.050 m, and a valid detection rate of 99.4% over 358 valid cycles out of 360 scheduled cycles. These results demonstrate the operational feasibility of the proposed RF-synchronized one-way ToF architecture for groundwater-level monitoring with reduced dependence on submerged pressure-sensing elements. In addition to the 12-month field validation, an extended range-dependent confined-tubular propagation test was conducted to evaluate the valid ToF detection capability at longer propagation distances. The results indicated stable valid detection up to 300 m under the tested tubular propagation condition, while detection quality decreased beyond this range due to reduced received ultrasonic amplitude, lower signal-to-noise ratio, acoustic attenuation, and increased timing uncertainty. Therefore, the 300 m result should be interpreted as a range-dependent valid-detection result under the tested confined tubular condition, not as a 12-month groundwater-depth validation over the full 300 m interval. The results also confirmed the importance of robust local estimation. The 10-shot median-like estimator reduced the influence of transient disturbances, RF timing jitter, weak ultrasonic arrivals, and occasional false detections compared with single-shot estimation. This confirms that repeated acquisition and ordered-sample processing are important components of the proposed duty-cycled monitoring architecture. The proposed method depends directly on the effective speed of sound in the well air column; therefore, temperature, humidity, airflow, pressure variation, and vertical air stratification may introduce depth error, especially at long propagation distances. The optional reference receiver should be interpreted as a local calibration aid rather than a guaranteed full-path compensation mechanism. In addition, the floating receiver is exposed to the internal well environment and is not a fully non-contact component in the strict physical sense. Its long-term survivability, axial alignment, tether stability, enclosure sealing, and resistance to condensation, corrosion, sediment, and biofouling require further multi-year evaluation.

Future work will focus on validation in wells with different diameters, casing materials, internal surface conditions, bends, obstacles, and airflow conditions. Further work will also include improved floating-unit centralization, standardized mechanical drawings, complete well-construction metadata, multi-point temperature/humidity compensation, raw signal logging, and longer durability testing. These improvements will help generalize the proposed method and clarify its practical applicability for large-scale groundwater-monitoring networks.

## Figures and Tables

**Table 1:** Hardware and installation specifications of the prototype system.

Subsystem	Component/Parameter	Specification Used in the Prototype
Surface unit	Main controller	Raspberry Pi-class single-board computer (Sony UK Technology Centre, Pencoed, South Wales)
	RF synchronization/communication	433 MHz ISM-band RF/LoRa transceiver (DFROBOT, Shanghai, China)
	Ultrasonic transmitter	40 kHz waterproof ultrasonic transmitter
	Ultrasonic excitation	Dedicated 12 V ultrasonic drive stage
	Power rails	3.3 V and 5 V logic rails; 12 V ultrasonic transmission rail
	Installation position	Mounted at the wellhead and oriented toward the well axis
Floating unit	Main controller	STM32F103C8T6 microcontroller (STMicroelectronics, Plan-les-Ouates, Switzerland)
	Ultrasonic receiver	40 kHz ultrasonic receiver with analog amplification/filtering front-end
	Receiver front-end	Adapted JSN-SR04T/AJ-SR04M-type ultrasonic receiving stage with custom timing control (Shenzhen Hong Shu Yuan Technology, Shenzhen, China)
	ToF capture	Hardware timer input capture after RF synchronization
	Local estimation	10 ToF samples per cycle; median-like estimator using central ordered samples
	Enclosure	Sealed protective housing for humid and water-exposed well environment
	Position	Floating at the groundwater surface inside the well
	Stabilization	Mechanically tethered to reduce lateral drift and maintain approximate axial alignment
Tether	Function	Passive mechanical stabilization and retrieval only; not used for signal transmission, power delivery, timing synchronization, electrical measurement, or depth estimation
Measurement geometry	Acoustic path	One-way airborne propagation from wellhead transmitter to floating receiver
Validation	Reference instrument	Calibrated electric water-level tape
	Reference datum	All estimated and manual depths referred to the same wellhead datum

**Table 2:** Test-well geometry, installation conditions, and stabilization parameters.

Parameter	Description/Value Used in the Experiment
Test site	Observation groundwater well, Tashkent foothill region, Uzbekistan
Well type	Vertical cased observation well
Maximum test depth	300 m
Groundwater-depth interval during validation	14–30 m below the wellhead datum
Observation-well casing/test pipeline inner diameter:	170 mm plastic pipeline
Casing wall thickness	5 mm
Surrounding soil/lithology	Foothill alluvial deposits consisting mainly of loam, sand, gravel, and fractured sedimentary layers
Wellhead datum	Fixed top-of-casing/wellhead reference point
Surface-unit position	Mounted at the wellhead
Ultrasonic transmitter position	Installed at the wellhead and oriented downward along the well axis
RF module position	Installed in the surface unit for synchronization and data communication
Floating-unit position	Floating at the groundwater surface inside the casing
Floating-unit function	RF synchronization reception, ultrasonic arrival detection, ToF estimation, and data return
Floating-unit enclosure	Sealed protective housing for humid and water-exposed conditions
Floating-unit stabilization	Mechanically tethered to reduce lateral drift and maintain approximate axial alignment
Passive mechanical tether-line function	Passive mechanical stabilization and retrieval only; not used for signal transmission, power delivery, timing synchronization, electrical measurement, or depth estimation
Maintenance inspection	Enclosure integrity, tether condition, receiver orientation, receiver-surface contamination, and approximate positioning checked during site visits
Reference measurement	Calibrated electric water-level tape
Reference datum	Same wellhead datum used for both manual and system measurements

**Table 3:** Comparative overview of water-level measurement technologies for observation wells.

Category	Reference	Principle	Range/Test Condition	Accuracy/Error
Pressure sensor	Solinst [32]	Submerged pressure sensing	100–200 m water column	±0.05% FS/model dependent
	Keller [33]	Submerged pressure sensing with barometric compensation	Up to ~100 m water column	±0.1% FS
Echo ultrasonic	Kang et al. [10]	Echo-based ultrasonic ToF	~1 m calibration range	0–20 mm
	Panagopoulos et al. [11]	Echo-based ultrasonic ranging	2–3 m open-channel test	2–5 cm
	Bresnahan et al. [34]	Echo-based ultrasonic ToF	~6.45 m coastal deployment	~5 cm
Echo acoustic well sensor	Eno Scientific [35]	Echo-based acoustic pulse	Up to 2100 m claimed range	~3 cm
Radar sensor	Catsamas et al. [16]	FMCW radar	2–3 m open-channel test	Depth-only MAE not reported
	Campbell Scientific [36]	Pulse radar	Up to 35 m open-surface range	±3 mm
Proposed method	Proposed RF-synchronized direct-ToF system	One-way ultrasonic ToF to floating receiver	300 m confined tubular test; 12-month field validation	MAE = 0.048 m; MaxAE = 0.050 m

**Table 4:** Monthly validation at the Tashkent foothills observation well depth below ground surface.

Month	$D_{m}^{ref}$ (m)	${\bar{D}}_{m}$ (m)	$SD_{m}$ (m)	$e_{m}$ (m)	Success Rate (%)	(N) (Cycles)
January	22.000	22.047	0.015	0.047	100.0	30
February	20.000	19.950	0.017	0.050	100.0	30
March	16.000	16.045	0.013	0.045	100.0	30
April	14.000	13.950	0.012	0.050	100.0	30
May	15.000	15.048	0.014	0.048	100.0	30
June	18.000	17.954	0.016	0.046	100.0	30
July	23.000	23.050	0.020	0.050	100.0	30
August	28.000	27.951	0.024	0.049	96.7	30
September	30.000	30.047	0.028	0.047	96.7	30
October	29.000	29.050	0.023	0.050	100.0	30
November	27.000	26.950	0.023	0.050	100.0	30
December	25.000	25.048	0.023	0.048	100.0	30

**Table 5:** Overall statistical agreement between the proposed system and reference measurements.

Metric	Value	Unit
Number of validation months	12	months
Total attempted cycles	360	cycles
Total valid cycles	358	cycles
Total invalid cycles	2	cycles
Overall valid detection rate	99.4	%
Mean reference depth	22.25	m
Mean estimated depth	22.26	m
Mean absolute error (MAE)	0.0483	m
Root mean square error (RMSE)	0.0484	m
Mean bias error (MBE)	+0.0075	m
Maximum absolute error (MaxAE)	0.0500	m
Mean absolute percentage error (MAPE)	0.231	%
Coefficient of determination (R^2^)	0.9999	—
Linear fit slope	1.0012	—
Linear fit intercept	−0.0198	m

**Table 6:** Representative raw ToF samples from one 10-shot measurement cycle.

Sample Index	1	2	3	4	5	6	7	8	9	10
Raw ToF sample (ms)	64.05	64.08	64.06	64.09	64.07	64.10	64.06	64.08	64.07	64.58

**Table 7:** Operational modes for duty-cycled operation.

Mode ID	Sampling Interval	Scheduled Active Window Concept (Floating Unit)	Typical Wake-Up Sequence Within Window	Qualitative Autonomy Implication	Recommendation Use-Case
M1	10 min	Short listening window opens every 10 min; outside the window the unit remains in deep sleep	RF RX enabled → Symbol 1 wakes MCU and enables ultrasonic RX → Symbol 2 triggers measurement → 10-shot ToF + filtering → return to sleep	Lowest autonomy (highest energy use due to frequent wake-ups)	Rapid dynamics, stress periods (peak irrigation, pumping tests)
M2	30 min	Listening window opens every 30 min; deep sleep otherwise	Same as M1	Low–moderate autonomy	Operational monitoring with moderate temporal resolution
M3	1 h	Listening window opens hourly; deep sleep otherwise	Same as M1	Moderate autonomy	Standard monitoring where hourly granularity is sufficient
M4	6 h	Listening window opens every 6 h; deep sleep otherwise	Same as M1	High autonomy	Seasonal trend tracking; reduced maintenance visits
M5	12 h	Listening window opens twice per day; deep sleep otherwise	Same as M1	Very high autonomy	Long-term surveillance, remote locations with limited access
M6	1 day	Listening window opens once per day; deep sleep otherwise	Same as M1	Maximum autonomy (minimal duty-cycle)	Strategic monitoring networks; baseline groundwater regime assessment

**Table 8:** Range-dependent performance of the proposed direct-ToF system under confined tubular propagation conditions.

Test Distance (m)	Attempted Cycles	Valid Cycles	Valid Detection Rate (%)	Mean ToF (ms)	ToF SD (ms)	Mean Absolute Error (m)	Maximum Absolute Error (m)
50	30	30	100.0	145.8	0.6	0.012	0.018
100	30	30	100.0	291.5	0.9	0.018	0.026
150	30	30	100.0	437.3	1.2	0.024	0.032
200	30	30	100.0	583.1	1.6	0.031	0.040
250	30	30	100.0	728.9	2.1	0.039	0.047
300	30	30	100.0	874.6	2.6	0.048	0.050
325	30	28	93.3	947.5	3.4	0.056	0.068
350	30	26	86.7	1020.4	4.1	0.064	0.079

**Table 9:** Quantitative comparison of single-shot and multi-shot estimation strategies.

Metric	Single-Shot Estimation	10-Shot Arithmetic Mean	10-Shot Median-like Estimator
Number of evaluated cycles	358	358	358
Mean absolute error, MAE (m)	0.073	0.056	0.048
Root mean square error, RMSE (m)	0.081	0.061	0.048
Maximum absolute error, MaxAE (m)	0.124	0.086	0.050
Standard deviation of depth estimate (m)	0.041	0.031	0.023
Median absolute deviation (m)	0.036	0.027	0.020
Outlier count	19	7	2
Outlier rate (%)	5.3	2.0	0.6
Valid detection rate (%)	96.1	98.0	99.4

**Table 10:** System-level component analysis of the proposed groundwater monitoring architecture.

Configuration	RF Synchronization	Direct One-Way Ultrasonic ToF	10-Shot Estimator	Duty-Cycled Operation	MAE (m)	RMSE (m)	Detection Rate (%)	Main Observation
Full proposed system	Yes	Yes	Yes	Yes	0.048	0.048	99.4	Reference configuration
Without multi-shot estimator	Yes	Yes	No	Yes	0.073	0.081	96.1	Higher variability and more outliers
Without reference calibration	Yes	Yes	Yes	Yes	0.061	0.066	99.1	Increased systematic bias under environmental variation
Shortened duty-cycle recovery window	Yes	Yes	Yes	Yes	0.069	0.077	95.8	Greater transient instability after wake-up
Reflection-based baseline in the same well	Yes/No	No	No/optional	Yes/No	0.094	0.118	88.0	Stronger multipath sensitivity in tubular geometry

**Table 11:** Estimated depth error caused by uncompensated sound-speed variation.

Sound-Speed Error, ΔV (m/s)	Approximate Interpretation	Error at 22 m	Error at 300 m
0.1	Small calibration/environmental residual	0.006	0.087
0.5	Moderate environmental mismatch	0.032	0.437
0.6	Approximately 1 °C temperature error in dry air	0.039	0.525
1.0	Stronger temperature/humidity mismatch	0.064	0.875
3.0	Approximately 5 °C temperature difference	0.192	2.624

## Data Availability

The data are contained within the article.
